# Welfare Impact of Carbon Dioxide Euthanasia on Laboratory Mice and Rats: A Systematic Review

**DOI:** 10.3389/fvets.2020.00411

**Published:** 2020-07-22

**Authors:** Patricia V. Turner, Debra L. Hickman, Judith van Luijk, Merel Ritskes-Hoitinga, Jan M. Sargeant, T. Miki Kurosawa, Takashi Agui, Vera Baumans, Woo Sung Choi, Yang-Kyu Choi, Paul A. Flecknell, Byeong H. Lee, Pedro J. Otaegui, Kathleen R. Pritchett-Corning, Keisuke Shimada

**Affiliations:** ^1^Department of Pathobiology, University of Guelph, Guelph, ON, Canada; ^2^Global Animal Welfare and Training, Charles River, Wilmington, MA, United States; ^3^Laboratory Animal Resource Center, School of Medicine, Indiana University, Indianapolis, IN, United States; ^4^Department of Health Evidence, SYstematic Review Center for Laboratory Experimentation (SYRCLE), Radboud University, Nijmegen, Netherlands; ^5^Department of Population Medicine, University of Guelph, Guelph, ON, Canada; ^6^Centre for Public Health and Zoonoses, University of Guelph, Guelph, ON, Canada; ^7^Faculty of Veterinary Medicine, Kagoshima University, Kagoshima, Japan; ^8^Department of Applied Veterinary Science, Faculty of Veterinary Medicine, Hokkaido University, Sapporo, Japan; ^9^Department of Animals, Science and Society, Utrecht University, Utrecht, Netherlands; ^10^National New Drug Development Cluster, Woojung Bio, Suwon-si, South Korea; ^11^Department of Laboratory Animal Medicine, College of Veterinary Medicine, Konkuk University, Seoul, South Korea; ^12^Institute of Neuroscience, Newcastle University, Newcastle upon Tyne, United Kingdom; ^13^Osong Medical Innovation Foundation, Cheongju, South Korea; ^14^Laboratory Animal Facilities, Autonomous University of Barcelona, Barcelona, Spain; ^15^Office of Animal Resources, Harvard University Faculty of Arts and Sciences, Cambridge, MA, United States; ^16^Department of Comparative Medicine, University of Washington, Seattle, WA, United States; ^17^Animal Resource Center for Infectious Diseases, Research Institute for Microbial Diseases, Osaka University, Osaka, Japan

**Keywords:** carbon dioxide, euthanasia, systematic review, mouse, rat, animal welfare, pain, distress

## Abstract

**Background:** There has been increased concern about the suitability of CO_2_ as a method for euthanasia of laboratory mice and rats, including the potential discomfort, pain or distress that animals may experience prior to loss of consciousness; time to loss of consciousness; best methods for use of CO_2_; and the availability of better alternatives. These discussions have been useful in providing new information, but have resulted in significant confusion regarding the acceptability of CO_2_ for rodent euthanasia. In some cases, researchers and veterinarians have become uncertain as to which techniques to recommend or use for euthanasia of laboratory mice and rats.

**Methods:** The International Association of Colleges of Laboratory Animal Medicine (IACLAM) convened a taskforce to examine the evidence for adverse welfare indicators in laboratory rats and mice undergoing CO_2_ euthanasia using a SYRCLE-registered systematic review protocol. Of 3,772 papers identified through a database search (PubMed, Web of Science, CAB Direct, Agricola, and grey literature) from 1900 to 2017, 37 studies were identified for detailed review (some including more than one species or age group), including 15 in adult mice, 21 in adult rats, and 5 in neonates of both species. Experiments or reports were excluded if they only assessed parameters other than those directly affecting animal welfare during CO_2_ induction and/or euthanasia.

**Results:** Study design and outcome measures were highly variable and there was an unclear to high risk of bias in many of the published studies. Changes in the outcome measures evaluated were inconsistent or poorly differentiated. It is likely that repeated exposures to carbon dioxide inhalation are aversive to adult rats and mice, based on avoidance behavior studies; however, this effect is largely indistinguishable from aversion induced by repeated exposures to other inhalant anesthetic gasses.

**Conclusion:** There is insufficient evidence to permit an unbiased assessment of the effect of CO_2_ inhalation during euthanasia on welfare indicators in laboratory mice and rats. Additional well-designed, unbiased, and adequately powered studies are needed to accurately assess the welfare of laboratory mice and rats undergoing euthanasia via CO_2_ gas.

## Introduction

Euthanasia, or provision of a good death, is considered a critical event in an animal's life and it is important for animal well-being that it be conducted in a humane manner. According to international guidelines on research animal euthanasia, a euthanasia procedure should result in rapid and irreversible loss of animal consciousness with minimal distress leading to eventual death (1). It should also be relatively easy, inexpensive, and safe for a trained operator to perform, be accessible to a wide range of possible users, and be esthetically acceptable to those performing or observing the procedure (1). Mice and rats commonly worked with in science and there has been significant interest in recent years in determining the best method(s) for euthanizing these animals in research settings (1). Humane methods are required for euthanizing infant rodents, juveniles, adults, and pregnant dams—as individual animals, in small groups, and sometimes in very large numbers. Research rodents are also euthanized because of spontaneous pain, sickness, injury, or deformity; because humane or experimental endpoints are reached; as a means of population management; and because of environmental emergencies (e.g., flooding or power failures) or depopulation needs (e.g., a biosecurity break). Given the wide variation of reasons for euthanasia, the methods must be safe for a range of different situations. Additionally, because animals are often euthanized at study end to harvest organs for additional investigations, the method of euthanasia should not interfere with the future use of tissues. A further consideration for euthanasia method is that it should not create any hazards for carcass disposal. For example, if cadavers are to be donated for feeding of zoo animals or captive wildlife then the cadavers must be free of substances that may adversely impact other animals. For all of these reasons, carbon dioxide (CO_2_) inhalation is the most common technique in use today for euthanasia of laboratory mice and rats (2, 3).

Narcosis following carbon dioxide gas exposure has been long recognized, and CO_2_ gas was used historically as a short term anesthetic agent for humans and animals for almost 200 years (3–5). Increasing concentrations of inhaled CO_2_ (hypercarbia) induces respiratory and cerebrospinal fluid acidosis, as well as cardiovascular depression, that eventually lead to stupor, with unconsciousness occurring at CO_2_ concentrations of ~15–20%. Prolonged exposure to high levels of CO_2_ (~40–50%) results in coma, apnea, hyperkalemia, and cardiac arrest (6, 7). While its use as an anesthetic agent in rodents has largely been discontinued for over two decades, CO_2_ gas inhalation is used widely around the world for research rodent euthanasia.

Recently, there has been concern that CO_2_ inhalation is inhumane as a method of euthanasia for laboratory rodents (8–10). Carbon dioxide is a normal component of inhaled air (0.04%) and internal sensors for CO_2_ levels drive respiration in most animals. Labored breathing and fear have been noted in mice when inhaled CO_2_ levels are ~5–15%. Fear is thought to be elicited via stimulation of an acid-sensing ion channel 1a located in the amygdala (11). Pain is thought to occur when conscious animals are exposed to high levels of CO_2_ (>47%) because of the carbonic anhydrase found within mucosal surfaces of the upper and lower respiratory tree, which converts CO_2_ to carbonic acid in the presence of water [reviewed in (9)]. This suggests that there is a potential for discomfort, distress, and/or pain to occur in mice and rats prior to the loss of consciousness when CO_2_ gas is used for euthanasia. However, there is conflicting information in published studies regarding the effects of CO_2_ gas inhalation on mice and rats during induction for euthanasia. This has resulted in uncertainty regarding the acceptability of inhaled CO_2_ for rodent euthanasia and uncertainty by veterinarians and others, such as researchers, regarding which techniques to recommend or use for euthanasia of laboratory mice and rats.

Because euthanasia is deemed a critical responsibility of the laboratory animal veterinarian (1, 12), the International Association of Colleges of Laboratory Animal Medicine (IACLAM; iaclam.org) identified a need to review the literature objectively to determine whether CO_2_ gas inhalation meets the definition of euthanasia (1), including evaluating criteria such as time to loss of consciousness and time to death; whether the method is suited to one or multiple animals; and further to compare cost, availability, operator safety, practicality of use in different circumstances, and esthetics of CO_2_ use on operators or observers. A task force of board certified laboratory animal veterinarians from around the world was convened to conduct a systematic review of the welfare impact (i.e., any behavioral or physiologic effects related to distress, aversion, and/or pain) of exposure to CO_2_ gas (alone or in combination with other agents) for euthanasia of neonatal and adult laboratory mice and rats.

## Materials and Methods

### Study Design and Systematic Review Protocol

A systematic review of the literature was conducted and reported using the PRISMA guidelines for reporting of systematic reviews [(13) and Supplementary Table 1]. The review protocol was evaluated and registered at http://www.syrcle.nl on May 3, 2017 (14).

### Search Strategy and Data Sources

On August 1, 2017, a systematic literature search was conducted concerning CO_2_ euthanasia of mice and rats using the following electronic databases: Medline (PubMed), Web of Science, CAB Direct, and Agricola (see Supplementary Table 2). No language or date restrictions were placed aside from database onset dates (Medline, 1950; Web of Science, 1900; CAB Direct, 1904; Agricola, 1970). A research librarian from the University of Guelph was consulted on the search strategy. In addition, reference lists were checked from all identified review articles on euthanasia of laboratory rodents as well as included studies for possible relevant references. An English-only grey (i.e., non-peer reviewed) literature search using Google Scholar was also performed for identifying possible graduate theses, study papers, and abstracts.

### Study Selection, Interventions, and Comparators

Primary reviewers (PT, DH, and TK) are board certified laboratory animal veterinarians and members of the IACLAM Taskforce on CO_2_ Euthanasia. For Phase 1 screening, one reviewer (PT) initially pre-screened all references based on title alone to remove obvious irrelevant references and all excluded reference titles were checked by two individuals (DH and TK). In cases of conflict, the title was retained for more detailed title and abstract screening. For Phase 2 screening, each reference was assessed by two independent reviewers [PT and (DH or TK)] based on the title and abstract with consensus agreement achieved for all retained papers. Studies were excluded if they were not an *in vivo* intervention (i.e., experimental or observational) study in mice or rats of any age, if CO_2_ inhalation or another inhalant-type anesthesia or euthanasia method was not evaluated, and if publications were from conference proceedings or only available in abstract form and would not permit further data extraction or assessment of risk of bias. Remaining papers were imported to EndNoteX7 (Clarivate Analytics, Philadelphia, PA, USA) and duplicates were removed. For Phase 3 screening, each full-text reference was assessed (PT and DH) using an online systematic review program (Distiller SR, Evidence Partners Inc., Ottawa, ON, Canada), and all conflicts were resolved by consensus. Studies were excluded if they met any of the original three exclusion criteria and/or the study did not measure any outcomes directly relevant to animal welfare or behavior.

### Data Extraction and Data Analysis

Data from studies meeting the study selection criteria were independently extracted by two reviewers (PT and DH) using a standardized form, which was tested on 3 pre-selected studies (JS). Discrepancies in data extraction were resolved by consensus.

Study level data included year published, country of origin of the work, authorship, and funding source. Population characteristics included rodent species (mouse, rat, or both), sex (only males, only females, or mixed sex populations), age group of rodents being studied (neonatal vs. post-weaned), stock or strain of the rodent models being evaluated, and whether animals had been used for gas exposure or anesthesia studies in the past. Study design characteristics included the number of experimental and control groups, the number of animals per group, and whether studies were single exposure euthanasia experiments or multiple exposure “anesthesia with recovery” experiments. Intervention characteristics included gas flow rates, duration of exposure, type of gas or euthanasia agent applied, concentration or ratio of gas in mixtures, and other euthanasia treatments. Only quantifiable outcome measures were included, and, where possible, mean (±SE) time of occurrence, number of occurrences, or change in the outcome measure were collected. If a study described pilot data together with definitive data only outcomes from the definitive studies were collected. Continuous outcome measures specifically collected included: heart rate, blood pressure, plasma corticosterone levels, time to loss of posture, time to loss of righting reflex, time to death, time to onset of labored breathing, and change in EEG activity, and dichotomous outcome measures collected included: increased c-fos expression in the brain, urination, defecation, vocalization, seizures, escape behaviors, increased activity on induction, and bleeding from the nose during induction. Individual studies varied in the definitions and methods used for many of these variables. Data were extracted from tables or graphs, if numerical data was not reported.

### Risk of Bias and Quality Assessment

Assessment of risk of bias and study quality were conducted independently by two reviewers (PT and DH) using the modified SYRCLE Risk of Bias tool (15) and any disagreements were resolved by consensus. The risk of bias tool was originally developed for studies with separate control and intervention groups. Only one study used separate non-treatment and comparison groups, thus risk of bias was evaluated for all single trial euthanasia-only studies in which there were two or more comparison groups. Reporting of animal randomization, blinding, and sample size calculations was also assessed for all reviewed papers as indicators of study quality.

## Results

### Study Selection and Characteristics

**Figure 1:**
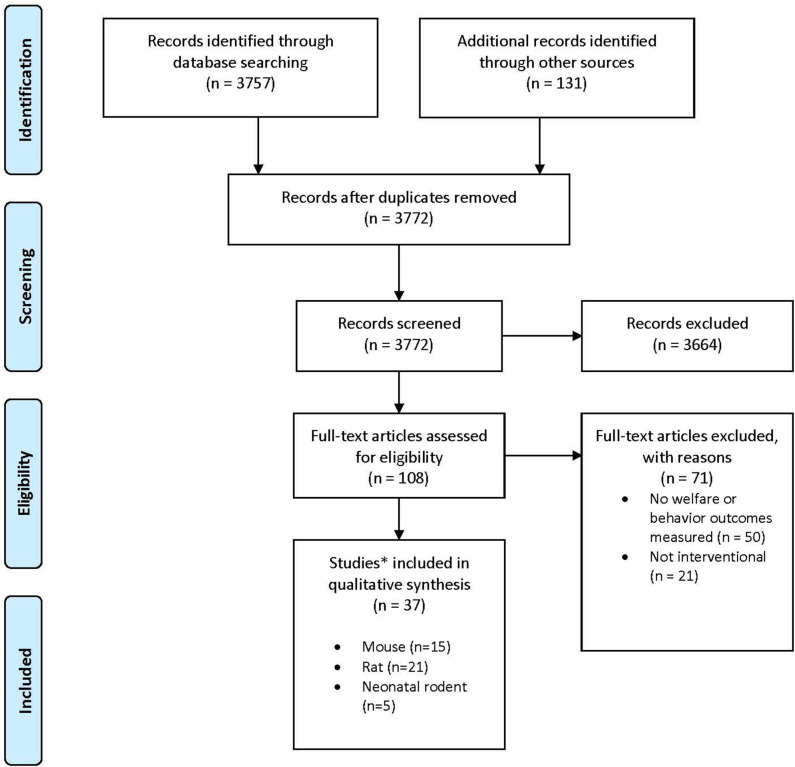
Preferred Reporting Items for Systematic Reviews and Meta-Analyses (PRISMA) study flow diagram [modified from (13)].

Results of the search strategy and study selection are presented in Figure 1. Of the 108 full text articles reviewed, 71 articles were excluded as they did not meet eligibility criteria and 37 articles were included. In total, 15 papers were reviewed for mice (16–30), 21 papers for rats (16, 22, 23, 31–48), and 5 papers were reviewed for neonatal rodents (21, 49–52), with three studies reporting results for both adult mice and rats (16, 22, 23) and one study reporting results for both adult female mice and their pups (21, 49–52). Not all studies evaluated euthanasia since welfare concerns in using inhalant gasses for euthanasia primarily revolve around what the animal experiences during the period from onset of exposure to the gas until loss of consciousness. Once an animal is deeply anesthetized, it is by definition insensible to pain and distress as long as the animal does not recover. Thus, some of the studies included in this review evaluated rodent behaviors, physiology, and time to induction with CO_2_ or other inhalant gases and then recovered animals for repeated exposures. Detailed study characteristics and findings are presented in Tables 1–3 for adult mice, adult rats, and neonatal rodents, respectively.

**Table 1:** Characteristics of the 15 adult mouse studies included in the systematic review.

**References**	**Strain, Sex, and Age**	**Intervention treatments of interest (group size)**	**Euthanasia or anesthesia induction with recovery**	**Frequency of exposure or re-use**	**Control intervention (group size)**	**Outcome measures (identification of effect)**
(16)	Quackenbush, females, mature	1. sublimation of dry ice 2. Chloroform into bedding 3. Ether-soaked cotton (*n* = 12)	Euthanasia	None, three replicates	Untreated controls in same environment in two densities, *n* = 1 and 3 Three replicates for each group	Chamber escape behaviors: ND LOP: shorter for CO_2_ Time to death: shorter for CO_2_
(17)	C57BL/6N, males, 16 week	1. CO_2_ 15% VDR 2. CO_2_ 30% VDR 3. CO_2_ 50% VDR 4. CO_2_ 100% VDR (*n* = 12–13)	Definitive: euthanasia	Re-used control mice in euthanasia studies	Pilot: air flow Controls: noise and air movement-only exposure, *n* = 28	Anxiety: ND Heart rate: ND between groups; blood pressure: ND LOP: faster as [CO_2_] increased Plasma cort: ND Time to death: decreased as [CO_2_] increased
(18)	C57BL/6NTac, males, 16 week	1. Isoflurane 5% in O_2_ 1 L/min then 100% CO_2_ after induction 2. Pentobarbital IP (*n* = 11–14)	Euthanasia	None	Used same control and CO_2_ comparator data as published in (17)	Anxiety: increased as [CO_2_] increased distance traveled: increased as [CO_2_] increased Heart rate: ND or increased as [CO_2_] increased Blood pressure: ND or decreased as [CO_2_] increased Plasma ACTH: reduced with pentobarb LOP: ND or faster as [CO_2_] increased Time to death: ND or faster as [CO_2_] increased
(19)	129 × S1/SVJ, mixed sex, 6–24 week	1. CO_2_ 100% facemask 2. CO_2_ 70% facemask 3. KCl 4. cervical dislocation 5. decapitation (*n* = 5–10)	euthanasia under succinyl choline	None	Each animal used as own control for baseline data	Time to loss of EEG signal: shorter for CO_2_ 100% = KCl = cervical dislocation = decapitation Visual evoked potential: reduced for CO_2_ 100%
(20)	NMRI, males, 12–16 week	1. CO_2_ 0.2 L/min FR 2. 8% sevoflurane in O_2_ 0.2 L/min FR 3. 4% isoflurane in O_2_ 0.2 L/min FR (*n* = 6–9)	Gas exposure with recovery	4 days chamber habituation, no gas	Air only central chamber, with two lateral chambers into which gas pumped, food restriction to maintain 85–95% pre-study weight, food pellets placed in lateral chambers during testing to motivate entry, compared combinations of gas exposures in three trials: sevo vs. CO_2_, CO_2_ vs. iso, iso vs. sevo	Approach/avoidance with food reward: time in sevo chamber increased compared with air only, ND between sevo vs. CO_2_ chambers, ND between time in iso vs. CO_2_ chambers, ND between time in iso vs. sevo chambers, ND in food reward eaten between three chambers
(21)	Mixed strains, cull genotypes, females, mature, and pregnant	1. CO_2_ 20% VDR 2. Pentobarbital IP 3. Cervical dislocation 4. Halothane in O_2_ 5. KCl under anesthesia 6. Cervical dislocation under anesthesia (*n* = 5–7)	Euthanasia or anesthesia induction with euthanasia	None	None, all compared to CO_2_	LOP: not consistently measured time to cardiac arrest: fastest KCl, next fastest CO_2_ = cervical dislocation (with or without anesthesia)
(22)	BALB/C, females, 9–15 weekk	1. CO_2_ 53% FR 2. argon 99% FR 3. CO_2_ 20%/argon 4.5% FR 4. CO_2_ 30%/argon 5.3% FR (*n* = 30 total) -high concentrations only	Definitive: gas exposure with recovery	Yes, up to 4 exposures per mouse over 6 week study	Pilot: gas level testing at low, medium, high, *n* = 30 (not re-used in definitive study) Definitive: 30 min chamber acclimation period, air-only 3 min control (each animal used as own control), measured time dwelling, time to withdrawal, and time to re-entry in chamber	Rearing: increased for CO_2_ 53% FR from baseline, ND between treatment groups approach/avoidance (time dwelling and time to withdrawal latencies): all [CO_2_] shorter vs. argon alone
(23)	BALB/C, females, 9–15 week	1. CO_2_ 50.8% FR 2. CO_2_ 34.9% FR 3. CO_2_ 25.5% FR 4. Argon 99.2% FR (*n* = 30 total) -high concentrations only	Definitive: gas exposure with recovery	Up to 7 exposures per mouse	Pilot: gas level testing at low, medium, high, *n* = 30 (not re-used in definitive study) Definitive: 30 min chamber acclimation period, air-only 3 min control (each animal used as own control), measured time dwelling, time to withdrawal, and time to re-entry in chamber	Anxiety/pain behaviors (urination, defecation, rearing): ND from baseline, ND between groups Approach avoidance latencies: time dwelling: shorter for all compared with air only, ND between groups; time to withdrawal latency: ND
(24)	CD1, males, surplus mature	1. CO_2_ 70% FR 2. AR 160% FR 3. CO 9% FR 4. isoflurane 3%/O_2_ 70% FR (*n* = 6–7)	Gas exposure with recovery	>50 exposures per mouse over 4 month study, three replicates per concentration	Control: air only, each animal as its own control -initial training to lower chamber with food reward -multiple CO_2_ concentrations evaluated	Approach/avoidance: latency to withdrawal decreased with increasing [CO_2_] (always left when chamber concentrations 13.5–18.2%), latency to withdraw for isoflurane = CO, latency to withdraw longest for argon
(25)	C57BL/6J-Tyr, females, 20 week	1. CO_2_ 50% FR 2. CO_2_ 40% FR 3. CO_2_ 30% FR 4. CO_2_ 20% FR (*n* = 5–6)	Euthanasia	None	None	Labored breathing: seen in all groups prior to LOP, ND seen between groups LOP: faster with increased [CO_2_] LORR: faster with increased [CO_2_]
(26)	C57BL/6J, males, 8 week	1. CO_2_ 20% VDR 2. isoflurane 5%/O_2_ 4 L/min 3. isoflurane 5% drop (*n* = 8–9)	Gas exposure with recovery	Two replicates	Three acclimation trials to test apparatus	Light/dark aversion pairing chamber latency: time to withdrawal: iso/O_2_ > CO_2_ = isoflurane drop, re-entry time: iso/O_2_ < CO_2_ = isoflurane drop; re-exposure: time to withdrawal reduced for iso/O_2_
(27)	C57BL/6J, males, 12–16 week	1. CO_2_ 20% VDR 2. isoflurane 5% in O_2_ 2 L/min (*n* = 6–7/group)	Euthanasia	None	None	LOP: ND LORR: faster with CO_2_ time to death: faster with CO_2_
(28)	CD1, retired sentinels, sex, and age not specified	1. CO_2_ 70% FR, 325 lux 2. CO_2_ 30% FR, 325 lux 3. isoflurane 3% then 70% CO_2_ FR after induction, 325 lux 4. CO_2_ 30% FR, 500 lux 5. CO_2_ 30% FR, 5 lux (*n* = 6)	Euthanasia	None, three replicates	Chamber light levels during euthanasia evaluated, euthanized in pairs in home cages, no acclimation period for lighting prior to euthanasia	Anxiety combined: highest for CO_2_ 70% = CO_2_ 70% + iso, increased at 500 vs. 40 lux = CO_2_ 30% 325 lux LOP: CO_2_ 70% < CO_2_ 70% + iso < CO_2_ 30% 325 lux Plasma cort: increased for CO_2_ 70% + iso
(29)	CD1, mixed sex, 10–12 week	1. CO_2_ 20% VDR 2. CO_2_ 20% + 60% N_2_O VDR 3. 20% CO_2_ + 60% N_2_ VDR (*n* = 3/sex/strain)	Definitive: euthanasia	Pilot: 3 × with 48 h washout between trial	Pilot: exposure with recovery, (*n* = 12 F)	Rearing: ND Jumping: increased for CO_2_ + N_2_ LORR: fastest for CO_2_ + N_2_O
(30)	CD1, females, 8–11 week	1. CO_2_ 100% VDR 2. CO_2_ 20% VDR 3. iso 5% in O_2_ 1.2 L/min then CO_2_ 100% VDR after induction 4. CO_2_ 20% VDR + ACE premed 5. CO_2_ 20% VDR + MDZ premed (*n* = 10)	Euthanasia	None	Exposure to CO_2_ gas for euthanasia with or without sedation	Increased activity: higher for iso Ultrasonic vocalization: increased for iso = CO_2_ 100% VDR Other behaviors: ND LORR: fastest for CO_2_ 100% VDR Plasma corticosterone: increased MDZ plasma ACTH: ND *c-fos*: decreased with CO_2_ 100% VDR Time to death: fastest for CO_2_ 100% VDR

*ACE, acepromazine; ACTH, adrenocorticotropin hormone; cort, corticosterone; EEG, electroencephalograph; FR, flow rate; IP, intraperitoneal; iso, isoflurane; KCl, potassium chloride; LOP, time to loss of posture; LORR, time to loss of righting reflex; MDZ, midazolam; ND, no difference; sevo, sevoflurane VDR, volume displacement rate*.

**Table 2:** Characteristics of the 21 adult rat studies included in the systematic review.

**References**	**Strain, Sex, and Age**	**Intervention treatments of interest (group size)**	**Euthanasia or anesthetic induction with recovery**	**Frequency of exposure or re-exposure**	**Control intervention (group size)**	**Outcome measure (identification of effect)**
(16)	Wistar, mixed sex, mature 220 g, and immature 88 g	1. Sublimation of dry ice 2. Chloroform into bedding 3. Ether-soaked cotton (*n* = 11 adult, *n* = 12 young)	Euthanasia	None, three replicates	Untreated controls in same environment in 2 densities, *n* = 1 and 3 (two for ether) -three replicates for each	Escape behaviors: ND Facial rubbing in young rats increased for ether LOP: fastest for CO_2_ Time to death: fastest for CO_2_
(31)	SD, males, 12–24 week	1. Isoflurane/O_2_ 2. Sevoflurane/O_2_ (*n* = 17 aversion-avoidance, *n* = 9 approach avoidance)	Anesthesia induction with recovery	Aversion-avoidance (light-dark), 3 times + training Approach avoidance, no. of training trials not specified	Used training sessions with O_2_ only as control	Aversion-avoidance latencies: ND, both aversive and increased with re-exposure Approach-avoidance latencies: ND, both aversive and increased with re-exposure
(32)	SD, males, 8–16 week	1. CO_2_ 10% VDR 2. Argon 50% VDR (*n* = 8)	Euthanasia	None	Control: each animal used as its own control with O_2_ only for baseline data	Rearing, sniffing, and ambulating: ND between groups or from baseline gasping and seizures: increased for argon only Ultrasonic vocalization: none recorded for any group Heart rate prior to LOP: faster for CO_2_ LOP: fastest for argon Time to death: fastest for argon
(33)	SD, females, 7–9 week	1. CO_2_ 30% VDR 2. isoflurane 2.5%/O_2_ (nine animals total, *n* = 3 CO_2_ only, *n* = 6 isoflurane and O_2_)	Repeated gas exposure with recovery	Not specified	Control: subset of animals exposed to O_2_ only (*n* = 6)	51 KHz ultrasonic vocalizations: increased as [CO_2_] increased, no vocalizations with isoflurane
(34)	SD, females, 250–500 g	1. CO_2_ 20% VDR 2. isoflurane 5%/O_2_ then 100% CO_2_ 3. CO_2_/O_2_ 70%/30% 4. pentobarbital (*n* = 8)	Euthanasia	None	None	LOP: ND LORR: fastest for 20% CO_2_ Heart rate: decreased as [CO_2_] increased Electromyelogram: ND Time to isoelectric electrocortigraph: faster with 20% CO_2_ Time to death: fastest for 20% CO_2_
(35)	Wistar, males, 32–52 week	1. CO_2_ 100% prefill 2. CO_2_ 22.5 L/min 3. CO_2_ 2.5 L/min 4. CO_2_ 22.5 L/min/O_2_ 11.25 L/min (*n* = 7)	Euthanasia	Used in sleep/wake study previously, details not provided	None	Gasping: increased with 100% CO_2_ prefill LOP: fastest for 100% CO_2_ prefill Heart rate: decreased with 100% CO_2_ prefill and fast flow CO_2_ Time to isoelectric EEG: fastest for 100% CO_2_ prefill
(36)	SD (3 different sources), sex not specified, 300–1,050 g	1. CO_2_ 100% prefill 2. CO_2_ 80% 3. CO_2_ 70% 4. CO_2_ 60% *n* = 6 in each of precharged and empty trials at each concentration	Definitive: euthanasia	Used in CO_2_ anesthesia pilot study	Pilot: anesthesia	Nasal bleeding and salivation prior to death: increased with 60% and 70% CO_2_ LOP: ND LORR: faster as [CO_2_] increased Time to death: faster as [CO_2_] increased
(37)	F344, males, 8 week	1. CO_2_ 6 L/min 2. Ace 7 mg + CO_2_ 6 L/min 3. Pentobarb 60 mg/kg + CO_2_ 6 L/min (*n* = 4)	Euthanasia	None	None, groups of animals sequentially killed by decapitation at three timepoints (30, 75, 120 s)	No urination, defection, vocalization or signs of pain in any group Increased tachypnea: all groups Serum glucose: ND Plasma [ACTH]: higher for pentobarb plasma [cort]: higher for pentobarb
(38)	SD, males, 32 week	1. CO_2_ 100% prefill, catheterized 2. CO_2_ 7.2 L/min FR, catheterized (*n* = 8)	Definitive: euthanasia with blood gas collection rats used in previous behavioral study, details not provided	Used in CO_2_ anesthesia pilot study and then carotid artery catheter placed Under anesthesia for definitive phase	Pilot: anesthesia	No distress observed for any group LOP: faster for 100% CO_2_ prefill LORR: faster for 100% CO_2_ prefill Time to death: faster for 100% CO_2_ prefill
(39)	SD, males, 300–350 g	1. CO_2_ 100% VDR 2. CO_2_ 30% VDR 3. CO_2_ 10% VDR (*n* = 7)	Euthanasia, radiotelemetry implantation under isoflurane anesthesia	None	Each animal used as its own control with air only for baseline data	No seizures noted in any animals gasping: increased for first 20 s with 100% CO_2_ Locomotor activity: ND from baseline LOP: faster for 100% CO_2_ Heart rate: decreased for all groups with time Blood pressure: decreased for all groups with time Time to death: faster for 100% CO_2_
(40)	Wistar, males, 40 week	1. CO_2_ 14.5% VDR 2. CO_2_ 70%/ O_2_ 30% 14% VDR 3. CO_2_ 70%/O_2_ 30% 21% VDR 4. Air (*n* = 8)	Anesthesia	Eight times in this study with four training trials Rats had been used multiple times in previous CO_2_ anesthesia studies, details not provided	Control: each animal used as its own control with air only for baseline data -initial training to lower chamber with food reward	Approach/avoidance: all gas treatments reduced latency to stop eating, latency to leave chamber, and number of food rewards eaten compared with air
(22)	Wistar, females, 9–15 week	1. CO_2_ 53% FR 2. argon 99% FR 3. CO_2_ 20%/argon 4.5% FR 4. CO_2_ 30%/argon 5.3% FR (*n* = 30 total) -high concentrations only	Definitive: gas exposure with recovery	Yes, up to four exposures per rat over 6 week study	Pilot: gas level testing at low, medium, high, *n* = 30 (not re-used in definitive study) Definitive: 30 min chamber acclimation period, air-only 3 min control (each animal used as own control), measured time dwelling, time to withdrawal, and time to re-entry in chamber	Rearing behavior: ND between treatment groups and baseline grooming face and sniffing: increased from baseline variably approach/avoidance latencies time dwelling and time to withdrawal: shorter for all [CO_2_] vs. argon only
(23)	Wistar, females, 15 week	1. CO_2_ 50.8% FR 2. CO_2_ 34.9% FR 3. CO_2_ 25.5% FR 4. Argon 99.2% FR (*n* = 30 total) -high concentrations only	Definitive: gas exposure with recovery	Up to seven exposures per rat	Pilot: gas level testing at low, medium, high, *n* = 30 (not re-used in definitive study) Definitive: 30 min chamber acclimation period, air-only 3 min control (each animal used as own control), measured time dwelling, time to withdrawal, and time to re-entry in chamber	Anxiety/pain behaviors (urination, defecation, rearing): ND from baseline, ND between groups Approach avoidance: latency time dwelling: shorter for all compared with air only, ND between groups
(41)	Wistar, males, 44 week	1. Ar 239% 2. Ar 199% 3. Ar 159% 4. Ar 120% (*n* = 7)	Definitive: gas exposure with recovery	Multiple times in two phases of this study with air training trials (details not provided) Rats had been used multiple times in previous CO_2_ anesthesia studies, details not provided	Control: 32 control air trials (same animals)	Approach avoidance: number of cereal pieces eaten + latency to leave shorter with increased [Ar]
(42)	SD, males, 400–500 g	1. CO_2_ 17.25% VDR 2. Ar 17.25% VDR 3. air 17.25% VDR (*n* = 8)	Euthanasia (CO_2_ only)	Multiple, details not provided	Control: each animal was tested with air as a control	No head shaking in any animal Rearing, escape behaviors and vocalizations: increased with CO_2_ No LORR or death with Ar
(43)	Wistar, males, 400–500 g	1. CO_2_ 17% VDR 2. CO_2_ 20% 3. CO_2_ 15% 4. CO_2_ 10% 5. CO_2_ 5% 6. Ar 90% (*n* = 9)	Gas exposure with recovery	seven times with 19 additional air/CO_2_ training trials	Control: each animal was tested with air as a control	Approach-avoidance: number of cereal pieces eaten + latency to leave decreased with increasing [CO_2_], no rats ate cereal and time dwelling only 3 s for Ar trials
(44)	Wistar, males, 20–52 week	1. CO_2_ 17% VDR (data used from previous trial) 2. peppermint in air 17% VDR (*n* = 7–9)	Gas exposure with recovery	>8 times -rats had also been used multiple times in previous CO_2_ anesthesia studies, details not provided	Control: each animal was tested with air as a control	Rearing and nose to lid: ND Escape behaviors: increased from baseline with increasing [CO_2_] Approach-avoidance: number of cereal pieces eaten + latency to leave decreased with increasing [CO_2_]
(45)	Wistar, males, 32 week	1. CO_2_ 27% VDR 2. CO_2_ 20% VDR 3. CO_2_ 14% VDR 4. CO_2_ 7% VDR 5. CO_2_ 3% VDR (*n* = 8)	Gas exposure with recovery	At least 10 times -rats had also been used multiple times in previous CO_2_ anesthesia studies, details not provided	Control: each animal was tested with air as a control	Approach-avoidance: number of cereal pieces eaten + latency to leave decreased with increasing [CO_2_]
(46)	SD, males, 200–224 g	1. CO_2_ prefill 2. Ar prefill 3. N_2_ prefill 4. CO_2_ 30% 5. Air only (*n* = 5–7)	Definitive: euthanasia	Rats exposed to three gasses on 3 days for pilot study, animals re-used in definitive study	Control: air only controls (*n* = 9) but had been exposed to three gasses previously -rats instrumented for telemetry under ketamine/xylazine anesthesia	Rats exposed to Ar or N_2_ had muscle spasms/convulsions prior to LOP Heart rate: decreased for CO_2_ groups with time Blood pressure: decreased for CO_2_ groups with time LORR: fastest for CO_2_ prefill time to death: fastest for CO_2_ prefill
(47)	SD, males, 271–391 g	1. CO_2_ 100% prefill 2. CO_2_ gradual fill (*n* = 4–5)	Euthanasia	None	Rats instrumented with transducers for heart rate and blood pressure under methohexitone	No vocalization or escape behaviors in either group All rats in CO_2_ prefill group urinated LOP: fastest for prefill CO_2_ Heart rate: decreased for CO_2_ groups with time Blood pressure: decreased for CO_2_ groups with time Time to death: fastest for CO_2_ prefill
(48)	SD, males, 8 week	1. CO_2_ 24% FR 2. isoflurane 5%/O_2_ -Experiment 2 only—at low vs. high light intensity levels (*n* = 8)	Gas exposure with recovery	None	Control: animals served as their own controls during training trials	Aversion to light vs. dark: more likely to stay in dark compartment until recumbent with single exposure to iso vs. CO_2_, more likely to leave isoflurane faster upon re-exposure

*ACE, acepromazine; ACTH, adrenocorticotropin hormone; cort, corticosterone; FR, flow rate; iso, isoflurane; LOP, time to loss of posture; LORR, time to loss of righting reflex; MDZ, midazolam; ND, no difference; pentobarb, pentobarbital; SD, Sprague-Dawley; VDR, volume displacement rate*.

**Table 3:** Characteristics of the five neonatal rodent studies included in the systematic review.

**References**	**Species, Strain, and Age**	**Intervention of interest (group size)**	**Outcome measures (identification of effect)**
(21)	Mouse, various, strains, fetal pups embryonic day 14–20 (see note), postnatal day (PND) 1–7, PND 8–14	1. CO_2_ 20% VDR 2. Na pentobarb IP, 800 mg/kg (PND 8–14 only) 3. Halothane 5%/O_2_ 0.4 L/min (PND 1–7 *n* = 11, PND 8–14 *n* = 15)	LORR: pentobarb faster than CO_2_ in both PND 1–7 and 8–14 pups Cardiac arrest: CO_2_ faster than pentobarb (evaluated only in PND 8–14 pups) Note: fetal mice (embryonic day 14–20) were also evaluated. Dams were euthanized with the three agents listed or via cervical dislocation with or without halothane anesthesia. For all methods, fetal pups were still alive at 20 min post-euthanasia/exposure of the dam.
(49)	Mice, various (including CD1, C57BL/6J, 129S2), ED 16	1. Cervical dislocation of dam 2. CO_2_ exposure of dam (details not specified) 3. Na pentobarb IP, 120 mg/kg to dam 4. Na pentobarb intraplacental, 600 mg/kg (*n* = 9)	Dam treatments—time to fetus death: pentobarb = CO_2_, sodium pentobarbital < cervical dislocation; no difference between dam strain Fetal treatment—time to fetal death: pentobarb intraplacental < pentobarb IP to dam = CO_2_ dam < cervical dislocation of dam; B6 = 129S2, B6 < CD1
(50)	Mice, various strains, PND 0–10	1. Plastic bag pre-filled with 100% CO_2_ (*n* = 5–10 per run, *n* = 2,355 total)	Time to death: 60 min for pups PND 0–6, 20 min for PND 7–10, recovery of pups after prolonged exposures common
(51)	Rat, SD and F344, PND 0–10	1. Plastic bag pre-filled with 100% CO_2_ (*n* = 10 per run, *n* = 791 total)	Time to death: 35 min for pups PND 0, 5 min for pups PND 10, recovery of pups after prolonged exposures common
(52)	Mice, C57BL/6 and CD1, PND 1–2	1. Plastic bags placed 0.5 mL isoflurane-soaked cotton into bag and resealed (*n* = 3–18, 76 total)	LORR 2 min, exposed for 30 min and then following 30–120 min post-exposure period 18/76 pups recovered

*IP, intraperitoneal; LORR, time to loss of righting reflex; pentobarb, pentobarbital; PND, postnatal day; VDR, volume displacement rate*.

There was considerable diversity in the methods used for exposure to CO_2_ and in all but one study, there was no attempt to use a separate control or sham-treated group that wasn't later exposed to a secondary euthanasia or anesthesia intervention within the same study. In no case did three or more studies from different laboratories use similar interventions, species, and outcome measures, or a separate control group, precluding combination of data for meta-analyses. An attempt to re-analyze outcome data was similarly unsuccessful because of the heterogeneity in interventions, outcome measures collected, and their timing.

### Risk of Bias and Quality Assessment

**Figure 2:**
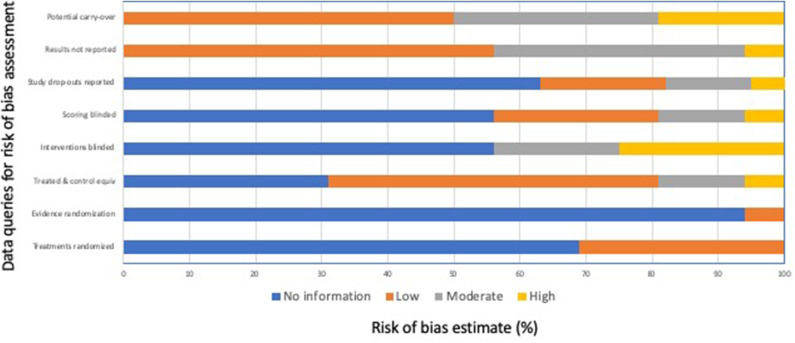
Risk of bias assessment summary for the 16 single trial studies in adult mice and rats, and rodent pups (16–19, 21, 25, 27, 28, 30, 32, 34, 35, 37, 39, 47, 49).

Nine of 15 mouse studies (16–19, 21, 25, 27, 28, 30), 7 of 21 rat studies (16, 32, 34, 35, 37, 39, 47), and two of five rodent pup studies (21, 49) (*n* = 16 separate studies as two evaluated both mice and rats of mice of different ages) were defined as single trial euthanasia studies and were assessed for risk of bias, although none included separate untreated and comparison groups. Other studies that were part of this review had incompatible study designs for the risk of bias assessment (i.e., single observation group, crossover studies, animals used in other gassing experiments, animals used in gas pilot studies prior to the definitive study, animals exposed to repeated subanesthetic gas concentrations or used repeatedly in induction-only trials). The results of the risk of bias assessment are summarized in Figure 2. In general, bias reduction measures were poorly reported by authors and were assessed as “no information.” In 11 of 16 studies, no information was provided about randomization of animals to different comparison groups. In the other five studies, randomization was mentioned; however only one of the studies provided actual evidence of randomization of some of their treatment groups. Sample size or power calculations were only mentioned in 1 of these 16 studies. In half of the 16 studies, animals served as their own controls; however, no information was provided in 5 of 16 studies, and in one study, a treatment group was added after all other portions of the study had been completed, such that no randomization or blinding could occur for this group. Most studies (9 of 16) did not provide information about blinding the treatments provided to animals or blinding those assessing the treatment effects. Because of study designs, treatments could not be blinded in 4 of 16 studies. For this reason, we interpreted both the risk of performance bias and the risk of detection bias to be moderate to high for most studies. No information about study drop-outs was provided in 10 of 16 studies, and this lack of reporting was deemed a moderate to high risk in 3 of 16 studies, in which study drop-outs were noted but not discussed further. In 7 of 16 studies, some results or methods were not fully reported. This included inconsistent reporting of results or methods, reporting of results in figures only (with or without error bars or confidence intervals), and not accounting for re-use of animals between different phases of the study. In 8 of 16 studies, a moderate to high risk for carry-over effects was identified due to re-use of animals between different phases of the study or use of a cross-over design.

**Figure 3:**
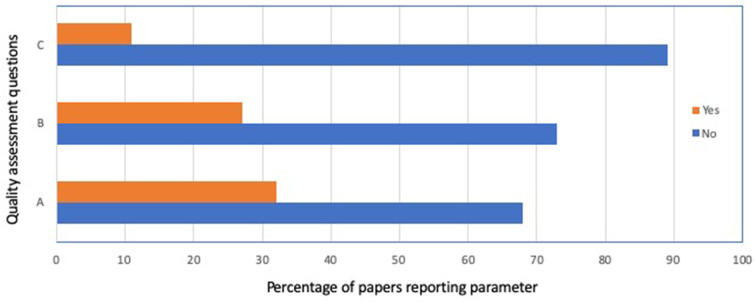
Quality assessment summary for the 37 papers included in this systematic review. (a) Reporting of any randomization, (b) reporting of any blinding, and (c) sample size calculation performed.

The overall quality assessment for the 37 papers included in this systematic review is shown in Figure 3. In general, randomization of animals to study groups was reported in 12 of 37 studies (32%), although, as mentioned above, only one paper provided actual evidence of randomization. Reporting of blinding for data collection or assessment occurred in 10 of 37 studies (27%), and reporting of a sample size calculation occurred in 4 of 37 studies (11%).

The risk of confirmation bias was determined to be high for some of the rat studies in which the same rats were re-used between studies conducted within one laboratory (anesthesia- or exposure-only type studies).

### Effects of Carbon Dioxide Inhalation on Adult Mice

The study characteristics for mice were remarkably diverse, emphasizing that comparisons were difficult to make. Of the 15 papers included for review for mice, one evaluated exposure to sublimation of dry ice (16), a technique no longer considered humane for rodent euthanasia because of the variability of CO_2_ gas production and the serious risk of freezing burns in animals if they touch the surface of dry ice (−78.5°C) (1). Eight studies (53%) evaluated outcomes in mice following a single exposure trial in which euthanasia resulted, while six studies (40%) evaluated outcomes following repeated exposures to CO_2_ or other inert or anesthetic gasses. When repeated exposures occurred, they ranged from 3 to 50 or more exposures, sometimes over a 4 month test period. Multiple strains of mice were evaluated across the different studies (although generally only one per study) in males (47%), females (33%), or both (20%) sexes of mice. Animals ranged in age from 8 weeks to over 24 weeks of age and studies sometimes were conducted opportunistically (2 of 15 studies), using animals remaining after other experiments conducted by other research groups had been completed. The nature of the previous work with the animals was generally not specified nor was the interval or time period always defined between the previous studies and the euthanasia/anesthesia studies. One study (7%) incorporated untreated controls into the study design, four studies (27%) had no control comparator group, and three studies (20%) used baseline values from the same animals as internal controls while seven (47%) induction-only studies used a variation of a cross-over designs in which mice were exposed to at least two or more gasses or inhalant agents, but not necessarily all tested agents.

Studies evaluated, tested, and reported exposures to CO_2_ and other inhalant gasses in a variety of ways. Exposure to CO_2_ or other inert or inhalant anesthetic gas was expressed as a percentage when delivered by face mask, and as volume displacement rate (VDR; 40% of studies) and/or as flow rate (FR; 40% of studies) when delivered into a chamber or cage. Volume displacement rate is considered the most accurate exposure method for calculating CO_2_ delivery into a chamber because of the marked variability in cage and chamber size (1). Because chamber dimensions were not routinely provided FR could not be converted to VDR. Similarly, gas flow patterns within the chamber will markedly affect the outcome following gas exposure, e.g., use of a gas diffuser and the specific location of gas introduction into the chamber, since all gasses studied are heavier than air. Some studies evaluated different VDRs or FRs of CO_2_, some compared one or more VDR/FR of CO_2_ with one or more inert or inhalant anesthetic gas and some studies compared one or more VDR/FR/percentage of CO_2_ with other physical or chemical methods of euthanasia, including pentobarbital sodium, potassium chloride, cervical dislocation, and decapitation. One study evaluated the effects of different VDRs of CO_2_ with or without premedication with acepromazine or midazolam (30) and one study evaluated the effects of different flow rates of CO_2_ when given with differing ambient light intensities (28). Flow or volume displacement rates of carbon dioxide evaluated ranged from 0.2 L/min to 15–100%.

A wide range of animal welfare-related outcome measures were reported, and the outcomes and direction of effect are listed in Table 1. When determining welfare impacts of exposure to CO_2_ or another method, the most important considerations are time to loss of consciousness and signs or changes suggestive of pain, discomfort or distress that are seen prior to loss of consciousness, since anything that occurs after this time will not be perceived by the animal. Assessment of unconsciousness was not conducted in the same way in studies that assessed this parameter. Loss of posture (LOP) or “nose down,” and loss of righting reflex (LORR) were all used by different research groups. At high flow rates, LOP occurs almost simultaneously with LORR; however, LOP can occur 5–20 s prior to LORR during induction for anesthesia. Assessment of LORR requires an experimental set-up that permits the observer to handle and manipulate the animal.

Of the 20 outcome measures evaluated in mice across the 15 studies, 10 were behavioral and included one or more of chamber escape attempts, “anxiety/pain” behaviors, jumping, increased activity, distance traveled, vocalization, rearing, urination, defecation, and labored breathing. One outcome measure was a trained response requiring repeated exposures to CO_2_ and other agents over training and definitive trials, that is, using approach-avoidance techniques. The remaining nine outcomes were physiologic in nature and included one or more of time to loss of posture or nose down, time to loss of righting reflex, heart rate, mean blood pressure, plasma corticosterone level, plasma ACTH level, time to loss of EEG signal, time to loss of visual evoked potential, and time to death.

In terms of behavioral findings, mice exposed to isoflurane demonstrated more movement in the chamber during induction than mice exposed to different VDRs of CO_2_, but there was no difference in escape attempts, pain or anxiety behaviors, including urination, defecation, and rearing, or ultrasonic vocalizations by mice induced with either CO_2_ or isoflurane (16–18, 22, 23, 28–30). In one study, labored breathing was noted in mice exposed to CO_2_ (20–50% FR) prior to LOP (25), but was not reported in a subsequent study by the same researchers using 20% VDR CO_2_ or isoflurane exposure (27).

In studies evaluating physiologic parameters in mice, time to LORR following CO_2_ exposure were consistently in three circumstances: when compared to other tested agents, such as isoflurane in oxygen (18, 27), for increasing VDRs or flow rates of CO_2_ (17, 28, 30), and when CO_2_ was combined with nitrous oxide (N_2_O) (29). Use of a sedative, such as acepromazine or midazolam, prior to administering CO_2_ did not shorten time to LORR (30). Heart rates and mean blood pressure increased from baseline prior to the onset of ataxia or LORR in all mice exposed to VDRs of CO_2_ between 15 and 100% and for mice exposed to isoflurane in oxygen (17, 18). There was no difference in peak heart rate or mean blood pressure with different VDRs of CO_2_ (15–100%), although, time to peak cardiovascular parameters was shorter with increasing VDRs of CO_2_ (17). Peak heart rates were higher for mice exposed to isoflurane compared with different VDRs of CO_2_, although peak mean blood pressure did not differ for mice exposed to CO_2_ vs. isoflurane (18). No differences were noted in plasma ACTH levels between mice exposed to different VDRs of CO_2_ vs. isoflurane (17, 18) and increased plasma corticosterone levels were noted for mice exposed to isoflurane for induction vs. CO_2_ alone (28). Time to loss of EEG signal and loss of visual evoked potential was fastest for mice exposed to 100% CO_2_ by facemask (vs. 70% CO_2_) (19).

Five studies did not examine time to loss of consciousness, anesthesia or euthanasia following exposure to CO_2_ or other inert or inhalant gasses, but instead evaluated effects of repeated exposure to different VDRs or FRs of CO_2_ in approach-avoidance studies. These results were sometimes compared to argon (alone or in combination with CO_2_), carbon monoxide (CO), isoflurane, and/or sevoflurane (20, 22–24, 26). VDRs, FRs, and percentage inhalant gas and rates of carrier oxygen were significantly different between these studies making comparisons difficult. In one study evaluating the impact of CO_2_ exposure (53% FR) compared to argon (99% FR) and mixtures of CO_2_ with argon, the time dwelling and time to withdrawal from chambers in which mice were exposed to gasses were shorter for mice exposed to CO_2_ alone or in combination with argon (22). In a subsequent study by this group comparing different FRs of CO_2_ (25.5–50.8% FR) alone to argon (99% FR), there was no difference in the chamber dwelling time and time to withdrawal from the chamber between any of the treatment groups (23). Guedes et al. (20) compared pairs of gasses (sevoflurane to CO_2_, sevoflurane to isoflurane, and isoflurane to CO_2_) and found no difference in dwelling time in the gas-only chambers between any of the pairs of gasses. When time to withdrawal from the chamber for CO_2_ (20% VDR) exposure was compared to isoflurane in oxygen vs. isoflurane drops on a cotton ball, withdrawal times were longest for isoflurane in oxygen and shorter for CO_2_ and isoflurane drops (26). However, upon re-exposure to the same agents, time to withdrawal from the chamber was shortest for isoflurane in oxygen (26). Finally, when mice were exposed 50 or more times to different gasses, including 70% FR CO_2_ vs. argon vs. CO vs. isoflurane, latency to withdraw from a chamber in which they received food rewards always occurred when CO_2_ chamber concentrations reached 13.5–18.2%, latency time to leave was equal for isoflurane and CO, and latencies were longest for argon (24).

### Effects of Carbon Dioxide Inhalation on Adult Rats

Similar to what was noted for mice, the study characteristics were highly diverse for rats. Of the 21 papers included for review for rats, one evaluated exposure to sublimation of dry ice (16), a procedure no longer considered acceptable, as mentioned above. This paper was not considered further. Ten studies (48%) evaluated outcomes in rats following a single definitive exposure trial in which euthanasia resulted (although in 3 of these studies, pilot studies involving gas exposures were also conducted using the same study animals), while another 10 studies (48%) evaluated outcomes following repeated exposures to CO_2_ or other inert or anesthetic gasses. When repeated exposures occurred, the number of prior exposures was often not specified. Eight of the studies (38% of the total) (31, 40–45, 48) were conducted by one lab and, as mentioned, many of the same rats were re-used in multiple studies with results published in separate papers. Studies were conducted largely in Wistar or Sprague-Dawley (SD) rats and in both sexes, although generally only one sex per study (71% were conducted in males only, 19% in females only, 5% in both sexes, and 5% did not specify animal sex). In these studies, animals ranged in age from 7 to 52 weeks. Similar to mice, details concerning previous experimental work with the rats was poorly specified and animals were used opportunistically in at least four studies. None of the rat studies incorporated untreated controls into the study design, four studies (19%) had no control comparator group, and 15 studies (71%) used baseline values from the same animals as internal controls and/or used the same animals in pilot trials. In seven of the euthanasia studies (33%), rats had been previously surgically instrumented with catheters or telemetry transducers under anesthesia (which included ketamine/xylazine, methohexitone, isoflurane, or sodium pentobarbital). Only one of these studies (34) re-exposed rats to the same anesthetic agent (isoflurane) during the definitive euthanasia trials.

Similar to studies in mice, a range of methods was used for reporting exposures to CO_2_ and other gasses. Some studies evaluated prefilled chambers of CO_2_ vs. different VDRs or FRs of CO_2_, some compared one or more VDR/FR of CO_2_ (sometimes mixed with oxygen or nitrogen) compared to one or more inert (e.g., argon, nitrogen) or inhalant anesthetic gas (isoflurane, sevoflurane), and one study compared one or more VDR or FR of CO_2_ with pentobarbital sodium euthanasia (34). One study evaluated the effects of different VDRs of CO_2_ with or without premedication with acepromazine (37). Flow or volume displacement rates of carbon dioxide evaluated ranged from 3% VDR to 100% CO_2_ chamber prefill.

A wide range of animal welfare-related outcome measures were reported, and the outcomes and direction of effect are listed in Table 2. For rat studies, assessment of unconsciousness was not conducted in the same way between studies, and both loss of posture (LOP) and loss of righting reflex (LORR) were used in different studies.

Of the 21 outcome measures evaluated in rats across the 21 studies, 10 were behavioral and included at least one of: chamber escape attempts, jumping/rearing, increased activity, distance traveled, vocalization, urination, defecation, labored breathing/gasping/head shaking, nasal bleeding, and seizures. One of the outcome measures assessed was a trained response requiring repeated exposures to CO_2_ and other agents over training and definitive trials using approach-avoidance techniques. The remaining 10 outcomes were physiologic in nature and included at least one of: time to LOP, time to LORR, heart rate, mean blood pressure, plasma corticosterone level, plasma ACTH level, serum glucose levels, time to isoelectric EEG signal, changes in electromyograph signal, and time to death.

For behavioral outcomes, several studies reported no adverse outcomes in rats (32, 38, 47), while other researchers reported nasal bleeding (36) or gasping (35, 39) at very high exposures of CO_2_ (60% FR or greater). Use of acepromazine as a sedative prior to administering CO_2_ did not provide any apparent extra benefit, as these researchers did not observe any urination, defecation, vocalization in rats exposed to CO_2_ at 6 L/min FR (37). Similarly, in the same study, tachypnea was seen in rats with increasing chamber concentrations of CO_2_, regardless of whether rats had been premedicated with acepromazine as a sedative (37).

In euthanasia studies evaluating physiologic responses, time to LORR, times were shortest for argon when supplied at 50% VDR or higher (32); however, seizures were reported in rats prior to loss of consciousness when very high exposures to argon (or nitrogen) occurred (46). Otherwise, LORR was fastest when rats were placed in novel chambers prefilled with 100% CO_2_ (35, 36, 38, 39, 46, 47). Only one of the euthanasia studies compared the effects of CO_2_ to isoflurane induction, followed by CO_2_ (34), and inductions (LORR) were noted to be slower with isoflurane compared with 20% CO_2_ VDR. Heart rates and mean blood pressure decreased from baseline in all rats exposed to VDRs of CO_2_ between 10 and 100% and for rats exposed to isoflurane in oxygen (32, 34, 35, 39, 46). In all studies in which EEGs were evaluated, time to isoelectric EEG and time to death were shortest for exposures to CO_2_ of 20% VDR or greater (34, 35, 39, 46, 47). In one study, exposure to argon at 50% VDR was compared to exposure to CO_2_ at 10% VDR, and time to death was faster for rats exposed to argon (32). No differences were noted in serum glucose in rats exposed to CO_2_ at 6 L/min when compared with rats exposed to the same level of CO_2_ but pre-treated with either acepromazine or pentobarbital; however, the rats exposed first to pentobarbital had higher serum levels of ACTH and corticosterone (37).

Ten studies reviewed did not examine time to loss of consciousness, anesthesia or euthanasia following exposure to CO_2_ or other inert or inhalant gasses, but instead evaluated effects of repeated exposure to different VDRs or FRs of CO_2_ or other inert or anesthetic gasses in approach-avoidance studies. These results were sometimes compared to argon (alone or in combination with CO_2_ and/or oxygen) or isoflurane and/or sevoflurane (22, 23, 31, 40, 41, 43–46, 48). VDRs, FRs, and percentage inhalant gas and rates of carrier oxygen were also significantly different between these studies making comparisons difficult. Similar to mice, in one study evaluating the impact of CO_2_ exposure (53% FR) compared to argon (99% FR) and mixtures of CO_2_ with argon, the dwelling time in the chamber and time to withdrawal from chamber in which rats were exposed to gasses were shorter for rats exposed to CO_2_ alone or in combination with argon (22). In a subsequent study by this group comparing different FRs of CO_2_ (25.5–50.8% FR) alone to argon (99% FR), there was no difference in the dwelling time and time to withdrawal between any of the treatment groups (23). When different flow rates of argon alone were compared, chamber dwelling time (i.e., latency to leave) decreased with increasing concentrations of argon (41). When aversion-avoidance was evaluated for isoflurane compared to sevoflurane, both agents were determined to be aversive and aversion increased with re-exposure (31). Further, when different CO_2_ VDRs or FRs were compared in an approach-avoidance paradigm, chamber dwelling times (i.e., latency to leave) were reduced as CO_2_ concentrations increased (40, 43, 45) or when compared to exposure to isoflurane (48).

### Effects of Carbon Dioxide Inhalation on Rodent Pups

When evaluating the effects of carbon dioxide inhalation on rodent pups, a variety of study designs and comparators were used. Five studies examined the effect of CO_2_ gas administration, isoflurane exposure, cervical dislocation, or sodium pentobarbital administered by intraperitoneal (IP) injection to the dam or by intraplacental injection (fetal mice only) on neonatal and fetal rodent pups. Four of the studies evaluated euthanasia of mouse pups (neonatal or embryonic) (21, 49, 50, 52) and one study evaluated the effects of CO_2_ gas exposure on neonatal rat pups (51). One study discussed the prolonged time to death of neonatal pups after exposure to halothane, a halogenated anesthetic no longer available in many countries (21). Multiple stocks and strains of mice were evaluated by the different neonatal mouse studies and SD stock and F344 strain were evaluated in the neonatal rat study.

The welfare outcomes measured for the rodent pups or fetuses for these studies included time to loss of righting reflex (LORR; pups only), time to cardiac arrest, and time to death (Table 3). Administration of sodium pentobarbital IP to postnatal day (PND) 8–14 mouse pups resulted in a faster LORR than for exposure of this same age group to CO_2_ gas; however, time to cardiac arrest was faster for CO_2_ gas exposure (21). Fetal mouse death was fastest following intraplacental administration of sodium pentobarbital compared to either administration of sodium pentobarbital IP to dams or exposure of dams to CO_2_ gas (49). Very young mouse pups (PND 0–6) of both sexes required prolonged exposures to CO_2_ to induce death (up to 60 min for PND 0)—if shorter times were used it was common for pups to recover when exposed to room air (50). Further, inbred strains were more resistant to the lethal effects of CO_2_ gas inhalation than outbred stocks (50). Exposure to high concentrations of isoflurane gas in oxygen for up to 30 min resulted in anesthesia of PND 1–2 mouse pups, but 24% of pups recovered after 30–120 min of room air exposure (52). Rat pups also required prolonged exposures to CO_2_ gas for euthanasia, although rats succumbed more quickly at PND 0 (35 min) than mice (60 min) (51). In summary, these findings indicate that very prolonged exposures are required when CO_2_ exposure is used for euthanasia of mouse or rat pups that are PND 6 or younger.

## Discussion

Death is a critical and permanent juncture in the course of any animal's life. Therefore, there is significant interest in ensuring that methods used for euthanasia of laboratory mice and rats are humane. Although many organizations have developed guidelines and recommendations for euthanasia of laboratory rodents [for example, (1, 53)], to date, these guidelines have not been based on a systematic review of the evidence available. This systematic review is the first to examine the evidence for the welfare impact of CO_2_ inhalation for euthanasia of laboratory mice and rats alone or in comparison with other euthanasia methods.

### Available Evidence and Quality

Studies evaluating the behavioral and physiologic effects of CO_2_ gas inhalation for euthanasia of laboratory mice, rats, and rodent pups were highly heterogenous in approach. Heterogeneity was caused by differences in the populations (sex of animal, different stocks and strains, prior use of animals in previous experiments, ages of adult animals), the interventions studied (CO_2_ gas; other anesthetic or inert gasses; physical methods; chemical methods; rate, method, and reporting of gas introduction and maintenance; age of perinatal rodent pups), and the outcome measures evaluated within and between studies. Most of the reported outcomes for single trial euthanasia studies in laboratory mice and rats showed no adverse effect of CO_2_ on behavioral or physiologic outcomes when administered to animals in a chamber at 70% VDR or less, and responses were generally no different from baseline. Evidence indicating discomfort prior to induction for euthanasia is present for CO_2_ gas when supplied to mice or rats in 100% prefilled chambers as gasping or tachypnea. Distress ultrasonic vocalizations (26.5 kHz) were also noted in mice during single trial euthanasia studies when exposed to CO_2_ in 100% prefilled chambers but not at lower VDRs. Similar distress ultrasonic vocalizations were noted when mice were induced with isoflurane in oxygen. When exposed to subanesthetic concentrations of CO_2_ gas over multiple training trials, mice and rats demonstrated avoidance behaviors in test trials. Avoidance behaviors were also seen in mice and rats exposed to subanesthetic concentrations of isoflurane and sevoflurane with multiple training trials. Reliability and overall interpretation of these findings is hampered by a number of limitations. In the one study in mice comparing CO_2_ euthanasia (100% prefill) to physical methods, the time to loss of EEG signal was similar between CO_2_, KCl injection, cervical dislocation, and decapitation.

Not all of the studies in the body of literature that are frequently cited as evidence of CO_2_ aversion evaluated physiologic or behavioral variables preceding euthanasia but rather evaluated parameters in response to exposures to subanesthetic CO_2_ or other gasses. Some of the studies reviewed evaluated behaviors that required repeated exposures to gas for reliable performance of a given behavior, and mice and rats in many studies were re-used between pilot and definitive phases, between different trials within the same study, and in some cases, between different experiments conducted within the same lab and published in separate papers. These repeat exposure experimental designs were likely used in an attempt to reduce the numbers of animals needed, an important 3Rs consideration. While repeat exposure studies can inform the research community about aspects of animal welfare, they cannot be considered as strictly equivalent to single trial euthanasia studies.

Appropriate reporting of methods is essential for interpreting study bias and quality in all biomedical research publications and to ensure reproducibility (54, 55). This has been emphasized repeatedly to the research community with publication of guidelines to support conduct and reporting of high quality experiments (56, 57). Our risk of bias assessment indicated generally poor or unclear bias in most of the studies for which this tool could be used. Furthermore, the overall study quality assessment suggested generally poor quality of evidence for the welfare effect of CO_2_ and other inhaled gasses for euthanasia of laboratory mice and rats, when considering sample size determination, and risks of performance and detection bias. Sample size calculations force research teams to define a primary outcome for their study *a priori* and the effect size needed to detect a difference between groups. When this is missing or poorly done, it becomes difficult to interpret the relevance of effects noted. For example, a statistically significant result within a small group of animals (e.g., a 5–10 s difference in loss of righting reflex between groups) may not be biologically relevant when the entire population is considered (58). Performance and detection bias are particularly critical for euthanasia procedures, in that euthanasia is a procedure that many people find distasteful and challenging to perform. Recently, a clear demonstration of bias occurred when study participants were asked to score the quality of observed loss of consciousness of mice and rats. The scoring differed when viewers were told that animals were being euthanized or anesthetized. (59). This demonstrates that it is essential that appropriate blinding occur when collecting and then assessing euthanasia outcomes.

Because of these general shortcomings in study design and conduct, the results of this systematic review indicate that there is insufficient evidence to permit an unbiased assessment of the impact of CO_2_ inhalation during euthanasia on welfare indicators in laboratory mice and rats. Further, while studies of repeated exposures to CO_2_ gas suggest that CO_2_ induction using prefilled containers may result in short periods of distress, induction with accepted inhalant anesthetic agents, including isoflurane and sevoflurane in oxygen, also are aversive to laboratory mice and rats. The aversion/avoidance differences between treatment groups are small under highly controlled laboratory conditions, and positive vs. negative differences in apparent aversion/avoidance between these agents and CO_2_ are not always clear cut. For example, repeated exposure to isoflurane was evaluated as being more aversive than repeated exposure to CO_2_ gas (26, 48, 60).

Further studies are needed to accurately assess the impact of inhalant euthanasia methods, such as exposure to CO_2_ gas on laboratory mice and mouse welfare, including the impact of euthanizing mice and rats in their home cages. A strategy for further research in this area has recently been published (61).

## Conclusion

There is insufficient evidence to permit an unbiased assessment of the overall impact of CO_2_ inhalation during euthanasia on welfare indicators in laboratory mice and rats. Additional well-designed, unbiased, and adequately powered studies are needed to accurately assess the welfare impact of CO_2_ gas euthanasia method for laboratory mice and rats and to identify alternative techniques that represent a significant improvement or benefit to animal welfare.

## Data Availability Statement

All datasets presented in this study are included in the article/Supplementary Material.

## Author Contributions

All authors contributed to conception and design of the study. PT, JL, and MR-H developed the SR protocol and submitted to SYRCLE for approval. PT, DH, and TK conducted the literature screening. PT and JS developed the data extraction questionnaire. PT and DH conducted the data extraction and ROB analysis. PT wrote the first draft of the manuscript. All authors contributed to manuscript revision, and read and approved the submitted version.

## Conflict of Interest

The authors declare that the research was conducted in the absence of any commercial or financial relationships that could be construed as a potential conflict of interest. The reviewer MRL declared a past collaboration with the author DH to the handling editor.

## Supplementary Material

Supplementary Table 1 PRISMA Checklist.

Supplementary Table 2 Comprehensive Search Strategy.
