# Supplementary material for: Welfare Impact of Carbon Dioxide Euthanasia on Laboratory Mice and Rats: A Systematic Review
Source: Front Vet Sci. 2020 Jul 22;7:411. doi: 10.3389/fvets.2020.00411 (PMC7387666; doi:10.3389/fvets.2020.00411)
Supplement: Supplementary Table 2 — Comprehensive Search Strategy. [file Data_Sheet_2.PDF]

Web of Science search Aug 1:

Component	Search string	Hits
CO2 (#1)	**TOPIC:** ("Carbon Dioxide" OR Carbon dioxide* OR CO2 OR CO 2 OR carbonic acid gas OR carbonic anhydride OR carbonic dioxide OR carbonic gas)	803,662
Euthanasia (#2)	**TOPIC:** ("Euthanasia" OR euthana* OR euthani* OR killing OR killed OR gassing OR gassed OR humane)	1,280,081
Animal (#3)	**TOPIC:** (murinae OR murine OR "mice" OR mouse OR mice OR "rats" OR rats OR rat OR "rodentia" OR rodentia OR rodent OR rodents OR "muridae" OR muridae OR "Animals, Laboratory" OR laboratory animal OR laboratory animals OR laboratory mammal OR laboratory mammals OR lab animal OR lab animals OR lab mammal OR lab mammals)	3,127,963
**Combined:**	**#1 AND #2 AND #3**	**2,898**

PubMed (Medline) search Aug 1

Component	Search string	records
CO2 (#1)	"Carbon Dioxide"[Mesh] OR Carbon dioxide*[tiab] OR CO2[tiab] OR CO 2[tiab] OR carbonic acid gas[tiab] OR carbonic anhydride[tiab] OR carbonic dioxide[tiab] OR carbonic gas[tiab]	**164929**
Euthanasia (#2)	"Euthanasia"[Mesh] OR euthana*[tiab] OR euthani*[tiab] OR killing[tiab] OR killed[tiab] OR gassing[tiab] OR gassed[tiab] OR humane[tiab]	**149849**
Animal (#3)	murinae[tiab] OR "mice"[MeSH Terms] OR mouse[tiab] OR mice[tiab] OR "rats"[MeSH Terms] OR rats[tiab] OR rat[tiab] OR "rodentia"[MeSH Terms] OR rodentia[tiab] OR rodent[tiab] OR rodents[tiab] OR "muridae"[MeSH Terms] OR muridae[tiab] OR "Animals, Laboratory"[Mesh] OR laboratory animal[tiab] OR laboratory animals[tiab] OR laboratory mammal[tiab] OR laboratory	**3226512**

	mammals[tiab] OR lab animal[tiab] OR lab animals[tiab] OR lab mammal[tiab] OR lab mammals[tiab]	
**Combined:**	**#1 AND #2 AND #3**	**747**

CAB Direct search Aug 1:

Component	Search string	Hits
CO2 (#1)	"Carbon Dioxide" OR Carbon dioxide* OR CO2 OR CO 2 OR carbonic acid gas OR carbonic anhydride OR carbonic dioxide OR carbonic gas	68
Euthanasia (#2)	"Euthanasia" OR euthana* OR euthani* OR killing OR killed OR gassing OR gassed OR humane	343,077
Animal (#3)	murinae OR murine OR "mice" OR mouse OR mice OR "rats" OR rats OR rat OR "rodentia" OR rodentia OR rodent OR rodents OR "muridae" OR muridae OR "Animals, Laboratory" OR laboratory animal OR laboratory animals OR laboratory mammal OR laboratory mammals OR lab animal OR lab animals OR lab mammal OR lab mammals	776,967
**Combined:**	**#1 AND #2 AND #3**	**1**

Agricola search Aug 1:

Component	Search string	Hits
CO2 (#1)	"Carbon Dioxide" OR Carbon dioxide* OR CO2 OR CO 2 OR carbonic acid gas OR carbonic anhydride OR carbonic dioxide OR carbonic gas	91,970
Euthanasia (#2)	"Euthanasia" OR euthana* OR euthani* OR killing OR killed OR gassing OR gassed OR humane	17,213
Animal (#3)	murinae OR murine OR "mice" OR mouse OR mice OR "rats" OR rats OR rat OR "rodentia" OR rodentia	176,512

	OR rodent OR rodents OR "muridae" OR muridae OR "Animals, Laboratory" OR laboratory animal OR laboratory animals OR laboratory mammal OR laboratory mammals OR lab animal OR lab animals OR lab mammal OR lab mammals	
**Combined:**	**#1 AND #2 AND #3**	**111**
